# Effect of an Antenatal Lifestyle Intervention on Dietary Inflammatory Index and Its Associations with Maternal and Fetal Outcomes: A Secondary Analysis of the PEARS Trial

**DOI:** 10.3390/nu13082798

**Published:** 2021-08-15

**Authors:** Sarah Louise Killeen, Catherine M. Phillips, Anna Delahunt, Cara A. Yelverton, Nitin Shivappa, James R. Hébert, Maria A. Kennelly, Martina Cronin, John Mehegan, Fionnuala M. McAuliffe

**Affiliations:** 1 UCD Perinatal Research Centre, School of Medicine, University College Dublin, National Maternity Hospital, Dublin 2, Ireland; sarah.louise.killeen@ucdconnect.ie (S.L.K.); anna.delahunt@ucdconnect.ie (A.D.); cara.yelverton@ucdconnect.ie (C.A.Y.); kennelly_maria@hotmail.com (M.A.K.); 2 School of Public Health, Physiotherapy and Sports Science, University College Dublin, Dublin 4, Ireland; catherine.phillips@ucd.ie (C.M.P.); john.mehegan@ucd.ie (J.M.); 3 Cancer Prevention and Control Program, Department of Epidemiology and Biostatistics, Arnold School of Public Health, University of South Carolina and Connecting Health Innovations LLC, Columbia, SC 29208, USA; nshivappa@chi-llc.net (N.S.); JHEBERT@mailbox.sc.edu (J.R.H.); 4 Department of Midwifery, National Maternity Hospital, Dublin 2, Ireland; mcronin@nmh.ie

**Keywords:** dietary inflammatory index, glycaemic index, intervention, antenatal, lifestyle, obesity, nutrition

## Abstract

We investigated the effect of an antenatal lifestyle intervention of a low-glycaemic index (GI) diet and physical activity on energy-adjusted dietary inflammatory index (E-DII^TM^) and explored its relationship with maternal and child health in women with overweight and obesity. This was a secondary analysis of 434 mother−child pairs from the Pregnancy Exercise and Nutrition Study (PEARS) trial in Dublin, Ireland. E-DII^TM^ scores were calculated for early (10–16 weeks) and late (28 weeks) pregnancy. Outcomes included lipids, inflammation markers, insulin resistance, mode of delivery, infant size, pre-eclampsia, and gestational diabetes. T-tests were used to assess changes in E-DII^TM^. Chi-square, correlations, and multiple regression were employed to investigate relationships with outcomes. The mean (SD) age of participants was 32.45 (4.29) years with median (IQR) BMI 28.25 (26.70, 31.34) kg/m^2^. There was no change in E-DII^TM^ in the controls (−0.14 (1.19) vs. −0.07 (1.09), *p* = 0.465) but E-DII^TM^ reduced by 10% after the intervention (0.01 (1.07) vs −0.75 (1.05), *p* < 0.001). No associations were found between early pregnancy E-DII^TM^ and maternal and child outcomes, except for increased odds of adverse cardiometabolic phenotype in women who delivered male (OR = 2.29, *p* = 0.010) but not female infants (OR = 0.99, *p* = 0.960). A low-GI antenatal intervention can reduce the inflammatory potential of diets. Sex differences should be explored further in future research.

## 1. Introduction

Women with overweight or obesity may enter pregnancy with higher baseline inflammation [1]. Evidence suggests the association between pre-pregnancy overweight and obesity and adverse maternal and foetal outcomes is mediated through inflammation [2]. Early pregnancy diet and weight may have implications for inflammation throughout pregnancy [3]. In addition, emerging evidence suggests that maternal inflammation during pregnancy may impact foetal neurodevelopment, possibly with long-term effects [4,5].

The dietary inflammatory index (DII^®^) was designed to quantify the inflammatory potential of the diet. Originally developed in 2009 and updated in 2014 [6,7], the DII^®^ is calculated using data on major categories of macronutrients, micronutrients, and flavonoids. Individual nutrients are scored for their inflammatory capacity based on a thorough review of the literature through 2010. The unique inflammatory effect of each of these nutrients has been quantified based on evidence for relationships with inflammatory markers such as C-Reactive Protein (CRP), Tissue-Necrosis Factor-Alpha (TNF-Alpha) and Interleukin-6 (IL-6) (6). This evidence is in non-pregnant populations, but the DII^®^ has been validated in pregnancy cohorts [3,8]. A benefit of the DII^®^ is that it can be universally applied internationally using data from any form of dietary assessment. Unlike other dietary indices such as the Healthy Eating Index (HEI), the Mediterranean diet score, or the Dietary Approaches to Stop Hypertension (DASH), it is based on absolute intakes rather than achievements of specific standards and guidelines. Energy-adjusted-DII^®^ (E-DII^TM^) considers the impact of caloric intake on overall dietary inflammatory potential and has better predictive ability compared to unadjusted DII^®^ [9]. As the E-DII^TM^ has improved explanatory ability compared to DII^®^, it is currently used as the definitive analysis in about 75% of all DII^®^-related papers [9].

There is growing interest in the role of the DII^®^ in health outcomes in pregnancy. In a variety of pregnancy cohorts, the DII^®^ or E-DII^TM^ has been associated with outcomes such as maternal inflammation, gestational diabetes mellitus (GDM), preterm birth, birthweight, and neonatal adiposity, particularly in women with overweight and obesity [8,10,11,12,13,14]. Furthermore, maternal DII^®^ associations with offspring childhood respiratory issues, emotional and behaviour symptoms, and body mass index (BMI) trajectories from birth to adolescence have been reported [15,16,17,18,19,20]. Evidence suggests higher BMI is associated with DII^®^ in adults and children [1,21]. Pre-pregnancy BMI is positively associated with pregnancy DII^®^ and both BMI and DII^®^ are associated with inflammation in pregnant women with overweight and obesity [1,3]. 

The limited available evidence suggests that a plant-based or Mediterranean diet may reduce DII^®^ scores [22,23,24]. There is, however, a paucity of evidence on strategies to reduce the inflammatory potential of the diet in pregnancy. To address this gap in the literature, we aimed to use data from the Pregnancy Exercise and nutrition Research Study (PEARS) randomised controlled trial (RCT) to assess the efficacy of an antenatal lifestyle intervention in reducing E-DII^TM^ scores in pregnant women with overweight and obesity. We also looked at the relationship between E-DII^TM^ score and maternal cardiometabolic health and pregnancy outcomes as a secondary aim. This will add to the current body of literature by focusing on a relatively homogenous group of women with low-risk, singleton pregnancies and raised BMI.

## 2. Materials and Methods

### 2.1. Study Sample

This is a secondary analysis of participants recruited as part of the PEARS study, which was conducted between March 2013 and August 2016 at the National Maternity Hospital in Dublin, Ireland. The study had institutional ethical approval from the National Maternity Hospital and written informed maternal consent. The PEARS study (ISRCTN registry, https://www.isrctn.com/ (accessed on 13 August 21), ISRCTN29316280) was an RCT of a mobile health (M-Health) behavioural lifestyle intervention with smartphone application (app) support to prevent GDM in pregnant women with overweight and obesity. Details of the study protocol and results have been published previously [25,26]. In brief, pregnant women (both nulliparous and multiparous) were invited to take part in the study at their first antenatal visit. Women were eligible if they were aged between 18–45 years, were between 10–15-weeks’ gestation and had a BMI of ≥25 kg/m^2^–39.9 kg/m^2^. They also needed to own a smart phone. Subjects were excluded if they had a multiple pregnancy, a medical disorder requiring treatment, GDM in a previous pregnancy, or previous poor obstetric outcome. Women were allocated into intervention or control (usual care) groups using computer-generated allocations in a ratio of 1:1. Women allocated to receive usual care were managed according to local and national guidelines; however, this does not include consistent nutritional, physical activity, or targeted gestational weight gain advice as standard [25]. The intervention involved a single education session at the start of their randomisation visit. The education was delivered by a research dietitian or nutritionist. The dietary information centred around achieving a low-GI diet and included additional advice on portion sizes of carbohydrates and general healthy eating for pregnancy recommendations. The education was equicaloric so did not promote weight loss, as the aim was to prevent GDM. An exercise prescription of 30 min of physical activity for five days a week was also given by an obstetrician. This information was re-enforced through a specifically designed smart-phone app, fortnightly emails and two face-to-face study visits, all underpinned by behaviour change theory. Previously published work using these data showed that the intervention group significantly reduced their glycaemic load (GL; a measure of how much a particular food or diet will increase an individual’s blood glucose level after consumption) and increased their exercise intensity compared to those who received usual care. This study used data from 434 out of the total 565 women who took part in the PEARS trial. This sample represents those who had dietary data in early pregnancy from which to calculate their E-DII^TM^ scores.

### 2.2. Data Collection

All women had their height and weight measured by a relevant healthcare professional at their first antenatal visit, which took place in early pregnancy at approximately 10–16 weeks’ gestation. Weight was measured to the nearest 0.1 kg in light clothing using a SECA weighing scale (SECA GmbH & co. kg., Hamburg, Germany). Height was measured to the nearest 0.1 cm using a wall-mounted stadiometer after removal of footwear. This was used to calculate BMI, an inclusion criterion for the study. Participants’ baseline visit took place approximately two weeks after their first antenatal visit. Demographic information collected at their first antenatal (baseline) visit included maternal age, ethnicity, parity, and smoking status. Economic advantage was assessed using the Pobal Haase-Pratschke (HP Pobal) Deprivation Index, a neighbourhood deprivation score based on Irish census data which considers the relative advantage or disadvantage of the mothers’ location of residence [27,28]. Data on blood pressure were extracted from antenatal medical records. Average systolic and diastolic blood pressure values in early pregnancy (10–16 weeks’ gestation) were calculated. 

Blood samples were collected at the baseline visit and the study follow-up (28 weeks pregnancy) after at least eight hours of an overnight fast. Cord blood samples were collected at delivery. At the shortest possible interval post venepuncture, blood serum samples were centrifuged at 3000 rpm for 10 min, and aliquots were stored at −80 °C pending analysis. Glucose was analysed using the AU680 Chemistry analyser (Beckman Coulter Inc., High Wycomb, UK) and the hexokinase method. Insulin and c-peptide were quantified by automated immune-assay (Roche Cobas 602; Roche Diagnostics, Basel, Switzerland) with typical CVs < 5%. Total cholesterol, high-density lipoprotein cholesterol (HDL-C) and triglycerides were analysed on a Roche Cobas 702 analyser (Roche Diagnostics). Low density lipoprotein cholesterol (LDL-C) was estimated using the Friedewald equation [29]. Concentrations of C3 complement were analysed according to the immunoturbidimetric assay for serum complement C3 (Rx Daytona; Randox Laboratories, Antrim, UK). Concentrations of CRP were analysed using a biochip array (Evidence Investigator™ Metabolic Syndrome Array II, Randox Laboratories, Antrim, UK).

### 2.3. Dietary Inflammatory Index 

Maternal dietary intakes were assessed in early pregnancy (14–16 weeks) and late pregnancy (28 weeks) through 3-day food diaries, including one weekend day. Women were asked to record all food and beverages consumed in their 3-day food diaries including the types and amount of food consumed. Volumes could be given in household measures (e.g., teaspoons, tablespoons) or actual weights. Data were entered into Nutritics Professional Nutrition Analysis Software, version 4.267, Research Edition (Nutritics, Dublin, Ireland, www.nutritics.com (accessed on 13 August 21)) by trained research nutritionists. Participants’ mean daily nutrient intakes, including macronutrients as percentages of total energy, were calculated at each time point using validated food composition databases [30,31]. The E-DII^TM^ was calculated by researchers at the University of South Carolina for early and late pregnancy using data on 27 macro and micronutrients for each participant, adjusted for energy intake. These are carbohydrate, protein, fat, alcohol, fibre, cholesterol, saturated fatty acids, mono-unsaturated fatty acids, poly-unsaturated fatty acids, omega-3 fatty acids, omega–6 fatty acids, trans-fat, niacin, thiamine, riboflavin, vitamin B-12, vitamin B-6, iron, magnesium, zinc, selenium, vitamin A, vitamin C, vitamin D, vitamin E, folic acid, and β-carotene [7]. In brief, a *z* score is developed for each energy-adjusted nutrient intake compared to an energy-adjusted global reference dataset [9]. These *z* scores are then converted to proportions, which are centred on zero by doubling and subtracting one; then, each is multiplied by the effect score which is the unique cumulative score provided to that nutrient based on the inflammatory potential identified in the literature. The results for each nutrient are combined to get an overall score reflecting the inflammatory potential of the diet [7]. Lower E-DII^TM^ values indicate a more anti-inflammatory diet while higher values indicate a more proinflammatory diet. The inflammatory potential of the diet therefore decreases with decreasing E-DII^TM^ scores [32]. Early pregnancy E-DII^TM^ scores were generated for 434 women. Of those, 290 women had late pregnancy E-DII^TM^ scores calculated.

### 2.4. Outcomes

There is no core outcome set for pregnancy nutrition research. However, our group is developing this through the PRENCOS study [33]. The Core Outcome Set for Studies on Obesity in Pregnant Patients (COSSOPP) is also relevant to this group [34]. The COSSOPP group have published findings from their systematic review [35] and qualitative interviews [36]. The systematic review revealed suboptimal reporting of foetal and neonatal outcomes in studies that included an intervention that could influence them. Maternal complications include incidence of GDM, pre-eclampsia, pregnancy-induced hypertension, and caesarean delivery. Diagnosis of GDM was identified at 28–30 weeks’ gestation using the criteria of the International association of Diabetes in Pregnancy Study [25]. We used the Edmonton Obesity Staging System (EOSS) to determine metabolic phenotype using clinical cut-offs from Canning et al., 2015 [37,38]. We categorised maternal cardiometabolic markers including total cholesterol, LDL cholesterol, HDL cholesterol, triglyceride concentrations, glucose, and blood pressure into low (stage 0) or some risk (stages ≥1). Different cut-offs have been used for the individual cardiometabolic markers in the EOSS, as detailed in the recent review by Atlantis et al., 2020 [39]. Women were given an EOSS score ≥1 if they met any of the following criteria: systolic blood pressure >130 mmHg, diastolic blood pressure >80 mmHg, fasting glucose ≥5.6 mmol/L, total cholesterol ≥5.2 mmol/L, LDL cholesterol >3.3 mmol/L, HDL cholesterol <1.6 mmol/L, and triglyceride ≥1.7 mmol/L. Data on neonatal outcomes were retrieved from medical records. Neonatal and birth outcomes include pre-term delivery using <37 and <34 weeks, small for gestational age (SGA) (birthweight <10th centile), large for gestational age (LGA) (birthweight >90th centile), macrosomia (birthweight >4000 g), low birth weight (<2500 g), ponderal index admission to neonatal intensive care unit, Apgar score <7 at five minutes, and congenital anomalies. The Gestation Network’s Bulk Calculator 6.2.3 UK was used to calculate birth weight centiles [26]. The ponderal/Rohrer index is a measure of leanness of a person as a relationship between mass and height. It is calculated by dividing weight in grams by height in cm^3^ [40].

### 2.5. Statistical Analysis

Categorical variables are presented as number and frequency (%). Continuous variables were assessed for normality through visual inspection of histograms, the Kolmogorov–Smirnov test for normality, and inspection of descriptive data including the mean and median. Continuous variables are presented as mean (standard deviation) or median and interquartile range (25th, 75th centile). All non-normally distributed data were log_10_ transformed for regression analysis or the appropriate non-parametric statistical tests were used (Spearman’s correlations or Mann−Whitney U test). Comparison statistics were generated through independent sample or paired-sample t-tests (with data split by study group). Chi-square (*χ*^2^) tests were used to compare categorical variables. Any analysis that was suggestive of an association (*p* < 0.05) was investigated in multiple regression models. The relationship between early pregnancy E-DII^TM^ and maternal and foetal outcomes was assessed using multiple linear and logistic regression with a forced entry approach for known potential confounders. The confounders were chosen a priori and included maternal age, maternal baseline BMI (≥30 kg/m^2^ yes/no), ethnicity (*White* yes/no), smoking (current smoker yes/no), maternal education (completed some third level), and study group (intervention/control). Variables to investigate the interaction effect of BMI and infant sex on the relationships between centred E-DII^TM^ and outcomes were also included in the models. In the case of a significant sex interaction effect, the analysis was run for males and females separately. In the case of a significant interaction effect of BMI, the analyses were run for women with overweight and obesity separately. We applied the Benjamini−Hochberg correction for multiple testing with a false discovery rate of 0.20, which is appropriate to support hypothesis generation. At first, *p* values < 0.05 were considered statistically significant. Values were then compared to their corresponding *q* value according to the Benjamini−Hochberg adjustment to determine significance. Statistical analysis was performed using IBM Statistical Package for Social Sciences software for Windows, version 26.0 (SPSS Inc, Chicago, IL, USA). All analyses were performed with pairwise deletion of missing variables.

## 3. Results

### 3.1. Demographic Variables

Table 1 includes the demographics of the cohort (*n* = 434). Median BMI was 28.25 (26.70, 31.34) kg/m^2^ and mean age was 32.45 (4.29) years. Approximately a third of the sample (32.5%) had obesity.

### 3.2. Dietary Inflammatory Index

Table 2 includes the results for E-DII^TM^ scores in the intervention and control groups. There was no difference in the E-DII^TM^ at baseline between intervention and control groups (*p* = 0.499, *q* = 0.125). The inflammatory potential of the diet reduced from early to late pregnancy in the intervention group (mean change −0.76 (1.15), *p* < 0.001, *q* = 0.005), but there was no change in the control group (*p* = 0.465, *q* = 0.116). After the study period, 20.4% of the intervention group had a more pro-inflammatory diet (E-DII^TM^ > 0) while the proportion of participants with this classification was over double (46.2%) in the control group (*p* < 0.001, *q* = 0.001).

### 3.3. Cardiometabolic Markers

Spearman and Pearson correlation analyses (Table 3) revealed a positive association between early E-DII^TM^ and early maternal concentrations of LDL cholesterol (*r* = 0.13, *p* = 0.011, *q* = 0.022), triglycerides (*r* = 0.11, *p* = 0.023, *q* = 0.031), insulin (*r* = 0.14, *p* = 0.004, *q* = 0.021), and C3 complement (*r* = 0.12, *p* = 0.039, *q* = 0.039) and a negative association with HDL cholesterol (*r* = −0.10, *p* = 0.039, *q* = 0.040). The associations with insulin (*r* = 0.13, *p* = 0.015, *q* = 0.028), triglycerides (*r* = 0.11, *p* = 0.042, *q* = 0.043), and HDL (*r* = −0.16, *p* = 0.002, *q* = 0.018) persisted into late pregnancy. E-DII^TM^ was positively associated with cord blood insulin (*r* = 0.15, *p* = 0.044, *q* = 0.046).

Table 4 includes the results of multiple linear regression models on the association between early E-DII^TM^ score and cardiometabolic markers. There was no potential interaction effect noted for either infant sex or BMI on these relationships (all *p* values > 0.05), except for a potential relationship between infant sex and E-DII on insulin in late pregnancy, *p* = 0.049, *q* = 0.124. When the data were stratified by infant sex, however, no significant associations were seen for E-DII in adjusted linear regression (all *p* > 0.05).

### 3.4. Pregnancy Outcomes 

We assessed the relationship between E-DII^TM^ score and maternal and foetal outcomes. In correlation analysis, no association was found between E-DII^TM^ and birthweight (*r* = 0.11, *p* = 0.816, *q* = 0.155), birth length (*r* = 0.50, *p* = 0.318, *q* = 0.078), head circumference (*r* = −0.00, *p* = 0.981, *q* = 0.193), placental weight (*r* = 0.03, *p* = 0.577, *q* = 0.161), ponderal index (*r* = −0.05, *p* = 0.310, *q* = 0.097), or gestational age (*r* = −0.02, *p* = 0.753, *q* = 0.175). Chi-square tests suggested a potential relationship between E-DII^TM^ and metabolic phenotype. More women with a proinflammatory diet (E-DII^TM^ > 0) were metabolically unhealthy according to the EOSS (107, 87.0%) versus women with an E-DII^TM^ < 0 (117, 76.5%), *p* = 0.026, *q* = 0.033). There were no other potentially significant associations between a proinflammatory diet and categorical variables including GDM (*p* = 0.614, *q* = 0.314), diagnosis of preeclampsia or pregnancy-induced hypertension (*p* = 0.673, *q* = 0.151), macrosomia (*p* = 0.810, *q* = 0.182), SGA (*p* = 0.491, *q* = 0.118), LGA (*p* = 0.465, *q* = 0.115), preterm birth (*p* = 0.323, *q* = 0.194), or mode of delivery (*p* = 0.272, *q* = 0.087). 

Table 5 includes the results of multiple logistic regression models examining the relationship between continuous early E-DII^TM^ score and categorical maternal and foetal outcomes. In most of the models, no suggestion of an interaction effect was seen for E-DII^TM^ or maternal BMI category or infant sex (all *p* values > 0.05). A potential interaction was seen for E-DII^TM^ and infant sex for the metabolically unhealthy phenotype (*p* = 0.047, *q* = 0.048). When analysed separately and adjusted for all confounders except infant sex, we found that each unit increase in early maternal E-DII^TM^ resulted in 2.29 increased odds of being metabolically unhealthy using the Edmonton Obesity Staging System in women who delivered a male (n = 137, OR = 2.29, *p* = 0.010, *q* = 0.025., 95% CI = 1.22, 4.31) but not female infant (n = 114, OR = 0.99, *p* = 0.960, *q* = 0.196, 95% CI = 0.63, 1.56). The sample was 52.3% male (Table 1). When stratified by infant sex in chi-square statistics, there was also significant relationship between a proinflammatory diet (E-DII^TM^ > 0) and metabolically unhealthy phenotype for male (*p* = 0.015, *q* = 0.028) but not female (*p* = 0.567, *q* = 0.066) infants. In this analysis, 79.3% (n = 65) of women with an anti-inflammatory diet (E-DII^TM^ < 0) had a metabolically unhealthy phenotype while a greater proportion, 93.7% (n = 59) of those with a proinflammatory diet (E-DII^TM^ > 0), was classified as metabolically unhealthy. There was a potential interaction effect of maternal BMI on E-DII^TM^ for mode of delivery (*p* = 0.033, *q* = 0.034); however, there was no significant relationship between E-DII^TM^ and mode of delivery when women with overweight (n = 261, OR = 0.85, *p* = 0.239 *q* = 0.073, 95% CI = 0.60, 1.23) and obesity (n = 122, OR = 0.62, *p* = 0.115, *q* = 0.061, 95% CI = 0.34, 1.12) were analysed separately in adjusted logistic regression. In chi-square statistics, there were no significant relationships between mode of delivery and E-DII^TM^ when stratified into women with overweight (*p* = 0.747, *q* = 0.173) and obesity (*p* = 0.123, *q* = 0.063) separately.

## 4. Discussion

This is the first study to assess the impact of an antenatal diet and exercise intervention on E-DII^TM^ in pregnant women. We found that the PEARS intervention reduced E-DII^TM^ from early to late pregnancy. No change was observed in the control group. As secondary analyses, we investigated the association between E-DII^TM^ score and markers of cardiometabolic health and pregnancy outcomes for both the mothers and offspring. While correlation and single-variable regression suggested a relationship between E-DII^TM^ and several maternal and foetal cardiometabolic health factors including insulin and HDL cholesterol, the associations were no longer significant after adjustment for multiple confounders and correction for multiple testing. Higher odds of adverse cardiometabolic phenotype were found with increasing E-DII^TM^ in women who delivered male infants, even after controlling for confounders and multiple testing.

Due to the growing body of evidence for the relationship between dietary inflammatory potential and pregnancy outcomes, strategies to reduce E-DII^TM^ in pregnancy are of great interest. The PEARS intervention included a diet and exercise prescription as part of an overall healthy lifestyle package. In a non-pregnant population of women with overweight and obesity, a diet or a combined diet and exercise intervention aimed to induce weight loss also reduced E-DII^TM^ [41]. A multifaceted intervention including diet, exercise, and psychological counselling has also been shown to reduce adiposity and E-DII^TM^ in adolescents with obesity [42]. Evidence suggests that having a low DII^®^ and being physically active is associated with reduced all-cause mortality [43]. 

In this study, E-DII^TM^ was reduced in the intervention group but did not change throughout gestation in the control group. This suggests that without an intervention, E-DII^TM^ is relatively stable throughout pregnancy. These findings are consistent with the limited available literature. In a longitudinal study of 49 women with overweight and obesity by Wallace et al., the E-DII^TM^ did not change from early to late pregnancy [3]. The PEARs antenatal lifestyle intervention was not designed to reduce inflammation but rather to reduce the glycaemic potential of the diet. The intervention included advice on reducing GI and GL during pregnancy such as swapping high GI foods for a lower GI alternative [25]. Using dietary data collected from 110 college students attending a rural public college in Louisiana, USA, Kim et al. found that GI but not GL was associated with the DII^®^ [44]. The PEARS intervention also advised on healthy eating recommendations for pregnancy. It is therefore possible that improved diet quality contributed to reduced E-DII^TM^ scores post intervention. In an observational study of young adults, a lower DII^®^ was found to correlate with healthier scores on other dietary indices including the HEI, Alternative-HEI, and DASH indices. This should be an area for future research in pregnancy [45]. The PEARS lifestyle intervention also significantly altered dietary intakes of macro and micronutrients. This includes a reduction in absolute intakes of carbohydrate, free sugar intake, fat, saturated fat, sodium, and calcium [46]. 

A study by Turner-McGrievy et al. as part of the Inflammation Management Intervention (IMAGINE) advised non-pregnant adults to consume a predominantly plant-based diet, made up of fruits, vegetables, whole-grains, legumes, and spices (garlic, cumin, etc.), with the aim of reducing DII^®^ and systemic inflammation [22]. Three optional portions of fish were included in the intervention; however, participants were asked to exclude all meat and dairy and avoid refined foods such as sugar, flour, and oils. Over a three-month period, the DII^®^ reduced in the intervention compared to control group [22]. The mean DII^®^ score of their control group was −0.38 ± 2.56 after three months while the intervention group achieved a mean DII^®^ score of −2.66 ± 2.44. The intervention group also saw a significant reduction in CRP and circulating lipids with a dose−response effect [22]. In other studies, individuals consuming a plant-based diet such as a vegan, vegetarian, or Mediterranean diet have been shown to have lower DII^®^ scores compared to those consuming low-fat diets [23,24]. In a study by Sen et al. in the Project VIVA cohort, vegetables, fruit, whole-grains, fish, and eggs were negatively associated with DII^®^ score while sugar sweetened beverages were positively associated [8]. Zhang et al. found in a prospective cohort study of over 2000 pregnant women in China that those with the highest tertile of DII^®^ (most pro-inflammatory diet) had higher intakes of red meat and rice-wheat products and lower intakes of nuts, fruits, vegetables, fish, eggs, and beans [10]. Similarly, in an Irish cohort of adults (aged 50–69 years), those with a higher E-DII^TM^ score had lower intakes of fruits and vegetables and higher intakes of dairy, meat, fish, poultry, eggs, fats, and sugars [47].

We did not find any significant relationship between early E-DII^TM^ and pregnancy or birth outcomes. Evidence suggests that higher pre-pregnancy BMI is associated with a higher DII^®^ score [1]. The E-DII^TM^ scores in our cohort of women with overweight and obesity in early pregnancy were on average negative at −0.41 ± 1.1 (with a range of −2.94 to 2.42). It is possible that the absolute values of E-DII^TM^ in our study mothers limited the ability to detect associations with some pregnancy outcomes. In the study by McCullough et al. of 1057 mother−child pairs, the median maternal E-DII^TM^ (both pre-pregnancy and pregnancy timepoints included) was also negative at −1.37, but they had a much larger range of −5.00 to 4.96 [48]. Like our study, when using data from all mothers, they found no association between E-DII^TM^ values and maternal or foetal outcomes including birthweight, gestational age, SGA, LGA, and mode of delivery [48]. Additionally, Buxton et al. found that in a study of 1216 pregnant women in Mexico, E-DII^TM^ (range −4.10 to 4.59) was not associated with preterm birth [49]. McCullough et al. found significant relationships when the data was split by maternal BMI. With this approach, they found higher E-DII^TM^ predicted caesarean delivery in women with obesity but not overweight [48]. Similarly, we found a potential interaction effect of maternal BMI on the relationship between E-DII^TM^ and mode of delivery, but sub-analysis did not find any significant associations in either the women with overweight or obesity when controlled for all confounders. Maternal obesity increases the risk of many pregnancy complications and adverse birth outcomes [50]. Previously published work with data from the PEARS study, however, found that women with obesity did not have a significantly greater incidence of caesarean delivery compared to women with overweight [51]. In a separate analysis, the data from Project VIVA was used measure maternal DII^®^ rather than E-DII^TM^ in the second and third trimester, and it was found that higher values were associated with lower birthweight in mothers with obesity but not overweight [8]. 

Cardiometabolic health during pregnancy is important due to the longstanding implications it may have for the mother postpartum, as well as for the growing foetus [52]. In non-pregnant populations, increased inflammation has been associated with metabolic complications including dyslipidaemia, diabetes, and the metabolic syndrome [10]. The negative impact of pro-inflammatory diets on metabolic phenotype was recently shown in a non-pregnant population of 300 healthy adults with obesity [53]. In correlation analysis, we found relationships between early E-DII^TM^ and early concentrations of LDL cholesterol, HDL cholesterol, triglycerides, insulin, and C3 Complement protein; however, significance was lost after controlling for multiple confounders, including BMI, in the regression analysis. We found an interaction effect of infant sex on the relationship between E-DII^TM^ and metabolic phenotype. In the sub-analysis, higher E-DII^TM^ score increased the odds of being metabolically healthy by over two-fold, and a greater proportion of women who delivered male infants with a proinflammatory E-DII^TM^ had metabolically unhealthy phenotype compared to those with an anti-inflammatory value (E-DII^TM^ < 0). Rafferty et al., using data from the PEARS study, did not find a relationship between infant sex and maternal early pregnancy cardiometabolic markers alone, suggesting a unique interaction in relation to E-DII^TM^ [51]. In the ALPHABET study, gender differences were also observed. Specifically, in an analysis with 4199 mother−infant pairs from two cohorts (early postnatal determinants of child health and development (EDEN) and the Southampton Women’s Study (SWS)), higher pre-pregnancy E-DII^TM^ was associated with lower birthweight, head circumference, birth length, and higher risk of small-for-gestational-age birth in male but not female infants [13]. Only the association with birth length and SGA remained when the E-DII^TM^ from pregnancy, rather than pre-pregnancy, and both sexes were included from all seven pregnancy cohorts (23,993 mother−child pairs). We did not find an interaction effect for infant sex on these outcomes and our findings are therefore in contrast with this recently published work by Chen et al. [13]. The average birthweights in some of the cohorts included in that individual participant data meta-analysis were greater than in the PEARS study [13]. The study also included women of all BMI categories (BMI 23.3 ± 4.2 kg/m^2^) and the mean E-DII^TM^ was higher than ours at 0.2 ± 1.7. 

A strength of this study is the homogeneity of the study population, which allows us to investigate the relationship between E-DII^TM^ and outcomes in the context of healthy pregnant women with overweight and obesity. Many older studies used DII^®^ to assess outcomes. We used the E-DII^TM^, which represents a refinement compared to the DII^®^, as energy contributes to the inflammatory potential of the diet and in unadjusted values, lower absolute intakes may reduce DII^®^ [9]. We applied further criteria to adjust the statistical significance for multiple testing. The Benjamini−Hochberg correction for multiple testing is a more conservative approach than the Bonferroni method that provides considerable adjustment to control for the false discovery rate, appropriate for this study design [54]. The findings have clear clinical implications by providing evidence on safe approaches to improve the inflammatory potential of the diet in pregnancy. Limitations include the fact that the E-DII^TM^ in this study was calculated using self-reported data and as such, it is subject to error and misreporting [55]. The study is a secondary analysis of a previous randomised controlled trial and as such, it is likely not powered to find significant relationships with maternal and child outcomes. The sample size was limited to those which have enough data to calculate E-DII^TM^ in early pregnancy and is smaller than other similar studies in the literature [1,8,11,12,19]. 

## 5. Conclusions

Previous work highlights the potential role of higher E-DII^TM^ in the development of adverse health outcomes during pregnancy and beyond. Our novel study found that a healthy antenatal lifestyle intervention, which included low-GI advice, reduced E-DII^TM^ in pregnant women with overweight and obesity. When using data from the entire cohort, we did not find any significant associations between E-DII^TM^ and maternal or foetal outcomes, after controlling for confounders. This suggests that the predictive value of E-DII^TM^ for adverse outcomes in pregnant women with obesity may be of greater importance when compared to lower BMI categories, rather than within those with raised BMI. Future research investigating this hypothesis in a larger sample size is warranted. We did, however, see increased risk of adverse cardiometabolic phenotype in women who delivered male but not female infants. This suggests a potential role of infant sex in the relationship that warrants future study.

## Figures and Tables

**Table 1:** Maternal and foetal characteristics.

	*n*	Value
Age (years)	433	32.45 (4.29)
Body Mass Index (kg/m^2^) *	434	28.25 (26.70, 31.34)
Body Mass Index category (*n*, % obesity)	434	141, 32.50
Ethnicity (*n*, % *White*)	421	396, 94.1
Smoking (*n*, % current)	434	22, 5.10
Parity (*n*, % 1 or more)	434	194, 44.70
Socioeconomic status (*n*, % above average advantage)	434	310, 71.40
Study group (*n*, % intervention)	434	224, 51.60
E-DII^TM^ in early pregnancy	434	−0.10 (1.15)
E-DII^TM^ in late pregnancy	290	−0.413 (1.12)
Gestational age at delivery (days) *	419	283.00 (276.00, 289.00)
Maternal cardiometabolic and inflammatory markers in early pregnancy		
Total cholesterol (mmol/L)	398	5.39 (0.87)
LDL cholesterol (mmol/L)	398	3.21 (0.86)
HDL cholesterol (mmol/L)	398	1.52 (0.44)
Triglycerides (mmol/L) *	398	1.42 (1.07, 1.68)
Glucose (mmol/L)	382	4.50 (0.34)
C3 Complement (mg/dl) *	291	154.40 (141.59, 174.04)
C-reactive protein (mg/L) *	275	1.39 (0.64, 2.88)
Insulin (mmol/L) *	397	8.52 (6.45, 11.46)
C-peptide (Umol/L) *	391	1.41 (1.09, 1.75)
Maternal pregnancy outcomes		
Gestational diabetes (*n*, %)	394	57, 14.50
Pre-eclampsia or pregnancy-induced hypertension (*n*, %)	378	26, 6.90
Early pregnancy Edmonton Obesity Staging System score ≥ 1 (*n*, %)	276	224, 81.20
Infant characteristics		
Infant sex (*n*, % male)	411	215, 52.30
Birth weight (g)	422	3643.93 (526.89)
Low birth weight (*n*, % <2500 g)	422	9, 2.10
Macrosomia (*n*, % >4000 g)	422	99, 23.50
Small for gestational age (*n*, % <10th centile)	395	23, 5.80
Large for gestational age (*n*, % >90th centile)	395	47, 11.90
Placental weight (g)	363	665.94 (146.69)
Birth length (cm)	395	51.33 (2.17)
Ponderal index (cm^3^) *	399	2.70 (2.50, 2.92)
Head circumference (cm) *	385	35.10 (34.30, 36.00)
Foetal cardiometabolic and inflammatory markers		
Total cholesterol (mmol/L) *	193	1.76 (1.46, 2.00)
LDL cholesterol (mmol/L) *	193	0.86 (0.70, 1.08)
HDL cholesterol (mmol/L) *	193	0.54 (0.45, 0.70)
Triglycerides (mmol/L) *	193	0.53 (0.42, 0.74)
Glucose (mmol/L)	30	4.35 (0.86)
C3 Complement (mg/dl)	158	90.26 (18.11)
C-reactive Protein (mg/L) *	144	0.03 (0.02, 0.05)
Insulin (mmol/L) *	193	4.97 (2.48, 8.22)
C-peptide (Umol/L) *	203	0.12 (0.10, 0.59)
Birth outcomes		
Mode of delivery (% caesarean delivery)	422	112, 26.50
Preterm birth (*n*, % <37 weeks)	419	15, 3.60

Continuous data are presented as mean ± standard deviation unless * which is median (interquartile range). E-DII^TM^ is energy-adjusted dietary inflammatory index. HDL = high density lipoprotein, LDL = low density lipoprotein. Early refers to data collected between 14–16 weeks and late refers to data collected at 28 weeks’ gestation.

**Table 2:** E-DII^TM^ throughout gestation and impact of PEARs lifestyle intervention.

	Intervention		Control		*p* Value	*q* Value
	*n*	Mean (SD)	*n*	Mean (SD)		
Early pregnancy E-DII^TM^	224	−0.06 (1.11)	210	−0.14 (1.19)	0.499	0.125
Late pregnancy E-DII^TM^	147	−0.75 (1.05)	143	−0.07 (1.09)	<0.001	0.003
Mean change E-DII^TM^	147	−0.76 (1.15)	143	0.07 (1.21)	<0.001	0.005
Within group comparison		* *p* < 0.001 (*q* = 0.006)		* *p* = 0.465 (*q* = 0.116)		

Data presented as mean ± standard deviation. *p* values are derived from independent sample t-tests, except for * *p* values which represent paired-sample *t*-tests comparing early and late E-DII^®^ within the intervention and the control groups separately. E-DII^TM^ is energy-adjusted dietary inflammatory index. Early refers to data collected between 14–16 weeks and late refers to data collected at 28 weeks’ gestation. *q* values represent the level of significance to which each *p* value is compared to as part of the Benjamini−Hochberg adjustment.

**Table 3:** Correlations between E-DII^TM^ in early pregnancy and maternal and cord cardiometabolic markers.

	Maternal (Early)				Maternal (Late)				Cord			
	*n*	*r*	*p* Value	*q* Value	*n*	*r*	*p* Value	*q* Value	*n*	*r*	*p* Value	*q* Value
Total cholesterol (mmol/L)	398	0.01	0.070	0.054	362	0.01	0.872	0.183	193	0.07	0.362	0.099
LDL cholesterol (mmol/L)	398	0.13	0.011	0.022	362	0.06	0.279	0.082	193	0.07	0.330	0.090
HDL cholesterol (mmol/L)	398	−0.11	0.039	0.040	362	−0.16	0.002	0.018	193	0.00	0.974	0.200
Triglycerides (mmol/L) *	398	0.11	0.023	0.031	362	0.11	0.042	0.045	193	−0.03	0.679	0.143
Glucose (mmol/L)	382	−0.02	0.656	0.150	-				30	0.19	0.314	0.088
C3 Complement (mg/dl)	291	0.12	0.039	0.041	294	0.05	0.401	0.106	158	0.03	0.729	0.157
C-reactive Protein (mg/L) *	275	0.03	0.606	0.148	276	−0.030	0.620	0.138	144	0.01	0.910	0.185
Insulin (mmol/L) *	397	0.14	0.004	0.021	364	0.13	0.015	0.029	193	0.15	0.044	0.046
C-peptide (ng/mL) *	391	0.06	0.228	0.075	364	0.03	0.641	0.142	203	−0.02	0.747	0.158

Values are generated from Pearson’s or * Spearman’s correlation statistic. HDL = high density lipoprotein, LDL = low density lipoprotein. *q* values represent the level of significance to which each *p* value is compared to as part of the Benjamini−Hochberg adjustment.

**Table 4:** Multiple linear regression models for E-DII^®^ and cardiometabolic and inflammatory marker.

		Single Variable				Adjusted						
	*n*	B	*p* Value	*q* Value	95% CI	B	*p* Value	*q* Value	95% CI	*R*2 Adj	Model *p*	Model *q*
Early pregnancy												
LDL cholesterol (mmol/L)	398	0.13	0.011	0.024	0.02, 0.17	0.17	0.157	0.067	−0.03, 0.21	0.06	0.011	0.017
HDL cholesterol (mmol/L)	398	−0.11	0.023	0.042	−0.08, −0.06	−0.08	0.326	0.100	−0.09, 0.03	0.05	0.001	0.009
Triglycerides (mmol/L) *	398	0.11	0.027	0.060	−0.00, 0.03	−0.02	0.849	0.172	−0.02, 0.02	0.08	<0.001	0.010
C3 Complement (mg/dl)	291	0.12	0.039	0.037	0.15, 5.68	−0.01	0.901	0.182	−4.40, 3.87	0.11	<0.001	0.012
Insulin (mmol/L) *	397	0.16	0.001	0.017	0.01, 0.05	0.08	0.333	0.096	−0.01, 0.04	0.15	<0.001	0.013
Late pregnancy												
HDL cholesterol (mmol/L)	362	−0.16	0.002	0.016	−0.12, −0.03	−0.16	0.066	0.055	−0.15, 0.01	0.04	0.001	0.015
triglycerides (mmol/L)	362	0.11	0.042	0.045	0.02, 0.11	0.11	0.194	0.072	−0.03, 0.15	−0.00	0.462	0.106
Insulin (mmol/L) *	364	0.12	0.024	0.030	0.00, 0.04	0.02	0.775	0.164	−0.02, 0.03	0.16	<0.001	0.016
Cord blood												
Insulin (mmol/L) *	193	0.14	0.058	0.052	−0.00, 0.10	0.10	0.384	0.103	−0.05, 0.12	0.02	0.184	0.049

CI = confidence interval. Variables included in multiple regression analysis as potential covariates were maternal age at recruitment (years), ethnicity (Caucasian yes/no), economic advantage (yes/no), smoking (current yes/no), study group (intervention yes/no), and maternal BMI (over 30 kg/m^2^ yes or no). Additionally, the interaction effect of centred E-DII^TM^ with infant sex or maternal BMI category (over 30 kg/m^2^ yes or no) was included in each of the models. Log transformed data were used for all non-normally distributed values *. Standardised B values are reported. HDL = high density lipoprotein, LDL = low density lipoprotein, C3 = C3 Complement protein. *q* values represent the level of significance to which each *p* value is compared to as part of the Benjamini−Hochberg adjustment.

**Table 5:** Multiple logistic regression models for E-DII^TM^ associations with maternal and foetal outcomes.

		Single Variable				Adjusted			
	*n*	OR	*p* Value	*q* Value	95% CI	OR	*p* Value	*q* Value	95% CI
Maternal health									
Gestational diabetes mellitus	355	1.05	0.714	0.154	0.82, 1.34	0.86	0.515	0.122	0.55, 1.35
Metabolically unhealthy phenotype in early pregnancy	276	1.27	0.086	0.058	0.96, 1.68	0.91	0.707	0.152	0.56, 1.48
Pre-eclampsia or pregnancy-induced hypertension	378	0.87	0.436	0.113	0.61, 1.24	1.09	0.784	0.169	0.59, 2.03
Mode of delivery (caesarean delivery)	483	0.90	0.251	0.076	0.74, 1.08	0.89	0.494	0.119	0.64, 1.24
Neonatal health									
Macrosomia (>4000 g)	383	0.95	0.606	0.139	0.78, 1.16	0.78	0.159	0.069	0.55, 1.10
Small for gestational age (<10th centile)	359	0.93	0.677	0.145	0.64, 1.34	0.85	0.594	0.131	0.47, 1.54
Large for gestational age (>90th centile)	359	1.06	0.683	0.146	0.81, 1.38	1.12	0.663	0.142	0.68, 1.82
Preterm birth (<37 weeks)	381	0.77	0.265	0.079	0.49, 1.22	0.97	0.938	0.191	0.45, 2.11

CI, confidence interval. Variables included in multiple logistic regression analysis as potential covariates were maternal age at recruitment (years), ethnicity (Caucasian yes/no), economic advantage (yes/no), smoking (current yes/no), study group (intervention yes/no), and maternal BMI (over 30 kg/m^2^ yes or no). Additionally, the interaction effect of centred E-DII^®^ with infant sex or maternal BMI category (over 30 kg/m^2^ yes or no) was included in each of the models. Odds ratios (OR) are reported using the Exp B values. Metabolic phenotype was classified using the Edmonton Obesity Staging System. The interaction effect of E-DII^®^ and infant sex or maternal BMI category was included in each of the models. *q* values represent the level of significance to which each *p* value is compared to as part of the Benjamini−Hochberg adjustment.

## Data Availability

The data presented in this study are available on request from the corresponding author.
